# Determination of a novel parvovirus pathogen associated with massive mortality in adult tilapia

**DOI:** 10.1371/journal.ppat.1008765

**Published:** 2020-09-24

**Authors:** Wenzhi Liu, Yecheng Zhang, Jie Ma, Nan Jiang, Yuding Fan, Yong Zhou, Kenneth Cain, Meisheng Yi, Kuntong Jia, Hua Wen, Wei Liu, Wuxiang Guan, Lingbing Zeng

**Affiliations:** 1 Yangtze River Fisheries Research Institute, Chinese Academy of Fishery Sciences, Wuhan, Hubei, China; 2 Center for Emerging Infectious Diseases, Wuhan Institute of Virology, Chinese Academy of Sciences, Wuhan, China; 3 Department of Fish and Wildlife Sciences and the Aquaculture Research Institute, University of Idaho, Moscow, Idaho, United States of America; 4 Collaborative Innovation Center, Zhuhai Key Laboratory of Marine Bioresources and Environment, School of Marine Sciences, Sun Yat-sen University, Guangdong, China; Blood Systems Research Institute, UNITED STATES

## Abstract

Tilapia is one of the most important economic and fastest-growing species in aquaculture worldwide. In 2015, an epidemic associated with severe mortality occurred in adult tilapia in Hubei, China. The causative pathogen was identified as Tilapia parvovirus (TiPV) by virus isolation, electron microscopy, experimental challenge, *In situ* hybridization (ISH), indirect immunofluorescence (IFA), and viral gene sequencing. Electron microscopy revealed large numbers of parvovirus particles in the organs of diseased fish, including kidney, spleen, liver, heart, brain, gill, intestine, etc. The virions were spherical in shape, non-enveloped and approximately 30nm in diameter. The TiPV was isolated and propagated in tilapia brain cells (TiB) and induced a typical cytopathic effect (CPE) after 3 days post-infection (dpi). This virus was used to experimentally infect adult tilapia and clinical disease symptoms similar to those observed naturally were replicated. Additionally, the results of ISH and IFA showed positive signals in kidney and spleen tissues from TiPV-infected fish. To identify TiPV-specific sequences, the near complete genome of TiPV was obtained and determined to be 4269 bp in size. Phylogenetic analysis of the NS1 sequence revealed that TiPV is a novel parvovirus, forms a separate branch in proposed genus Chapparvovirus of *Parvoviridae*. Results presented here confirm that TiPV is a novel parvovirus pathogen that can cause massive mortality in adult tilapia. This provides a basis for the further studies to define the epidemiology, pathology, diagnosis, prevention and treatment of this emerging viral disease.

## Author summary

A novel parvovirus isolated from adult tilapia causes substantial morbidity and mortality. Using a SISPA-PCR and RACE, we identified and characterized 4269 nucleotides of this parvovirus. Tentatively named Tilapia parvovirus (TiPV), this is to our knowledge the first putative member of the family *Parvoviridae* shown to infect a teleost host. We found that a nucleotide sequence similarity search by BLASTX had no significant matches with other viruses, while amino acid sequence comparison indicated approximately 34.6% ~ 50.0% amino acids (aa) homology with other parvoviruses. Similarities between the genomes of parvoviruses infecting hosts in different phyla or divisions indicate a need to update previously suggested hypotheses on the origins of parvovirus. Our findings may represent new avenues to explain viral evolution and suggest a need to further study parvovirus pathogenesis.

## Introduction

Tilapia (*Oreochromis niloticus*) are native to Africa, belonging to the family *Cichlidae* of the order *Perciformes*, and are the third-ranking aquaculture species after finfish species within the *Cyprinidae* and *Salmonidae* families. Annual global production equals approximately 5.88 million metric tons with a current value in excess of $11 billion U.S. dollars (USD) [1–2]. Asia leads the world in production with 72% produced, primarily in China and Southeast Asia [1].

A wide range of bacteria, fungi, and parasite diseases have been described for tilapia aquaculture in China [3–5]. However, reports of viral infections associated with Chinese tilapia production are limited. In 2015, a massive mortality event was reported in cage-farmed tilapia in China and viral particles were observed to be associated with clinical disease signs [6]. The newly emerging disease developed rapidly and could reach 60–70% mortality. Oral administration of antibacterial drugs and water disinfection were not effective to control disease. *Dactylogyrus* and *Streptococcus agalactiae* are commonly found in tilapia, but they are not associated with the clinical features observed in this study, and moreover, attempts to identify it as a known viral agent, such as the herpes-like tilapia larvae encephalitis virus (TLEV) [7], the viral nervous necrosis (VNN) betanodavirus [8–9], iridovirus [10] and the Tilapia Lake virus (TiLV) [11–12] were unsuccessful. These results led us to suspect a novel viral agent probably existed.

The objective of this study was to fully characterize a parvovirus pathogen isolated from tilapia in China. Here we report results from virus isolation, electron microscopy, animal experiments, *In situ* hybridization (ISH), indirect immunofluorescence (IFA), PCR, and gene sequence analysis. This parvovirus pathogen (named TiPV) was isolated and determined to be the causative agent of an epidemic in tilapia in China.

## Results

### Clinical signs of the disease

**Fig 1:**
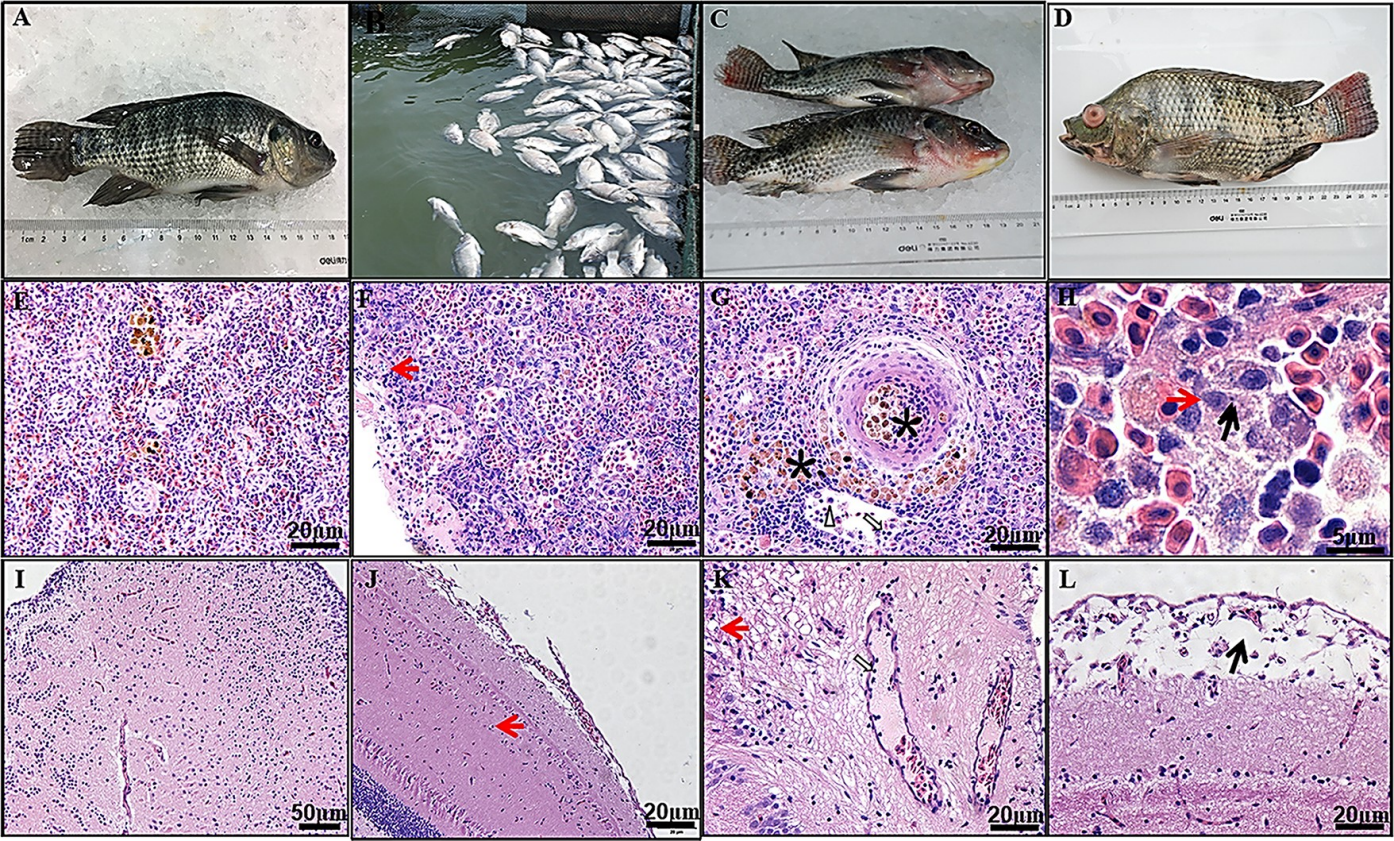
Outbreaks, clinical symptoms and pathological analysis. (A) Healthy tilapia; (B) Tilapia disease outbreak in cage-cultured results in massive mortality (August 2015; Jinmen, Hubei province, China); (C, D) Gross pathological signs of infected tilapia, including hemorrhages on the lower jaw, anterior abdominal and the fin bases, accompanying with exophthalmos eyes and pronounced ocular lesions. (E) Healthy spleen; (F) Moderated diseased spleen infected at 3^rd^ days after post TiPV-infection; (G, H) Severe diseased spleen infected at 5^th^ days after post TiPV-infection. lymphocytes (white arrow) and macrophages (white triangular arrowheads) in diseased spleen sinusoids, necrotic splenocytes (red arrow), virus inclusion body (black arrow) and melano-macrophage centers (asterisk) in affected spleen; (I) Healthy brain; (J) Moderated diseased brain infected at 3^rd^ days after post TiPV-infection, vacuolated neurons with marginated nuclear (red arrow); (K, L) Severe diseased brain infected at 5^th^ days after post TiPV-infection, vacuolated neurons with marginated nuclear (red arrow), lymphocytes (white arrow) in the blood vessel, edema of cerebral cortex (black arrow). HE staining. Bar = 20um (A, B, E, F), 50um (D), 5 um(C).

Initial disease outbreaks were reported in the cage-cultured adult tilapia from August to September 2015, resulting in high mortality (Fig 1B). Water temperature ranged from 25°C to 32°C at sites, and the most suitable temperature for the disease outbreak was determined to be between 28°C and 30°C. This disease appeared to be highly contagious and lethal to all sizes of cage-cultured adult tilapia and mortality reached between 60% and 70%. Clinical signs observed included lethargy, anorexia, and a change in swimming behavior that included darting or corkscrew movements. Compared to healthy fish (Fig 1A), diseased fish presented hemorrhages on the body surface, lower jaw, anterior abdomen, and fin bases, along with accompanied exophthalmia and pronounced ocular lesions (Fig 1C and 1D).

### Parasitology and bacteriology

Microscopic examination of fresh preparations from the gills revealed a minor infestation of *Dactylogyrus* sp. Bacterial isolation was attempted from the liver and kidney of moribund fish on BHI agar plates, and showed that the bacterial pathogen *Streptococcus agalactiae* was present.

### Histopathology

The histologic lesions of spleen, kidney, heart, brain and gill tissues were noted and summarized below:

Spleen: The necrotic splenic white pulp of diseased fish was infiltrated by many lymphocytes and macrophages (Fig 1G). More melano-macrophage centers were found in spleen parenchyma and blood vessels and adjacent to the area of necrosis (Fig 1G). The necrotic spleocytes exhibited a condensed and marginated nucleus and virus inclusion bodies in moderate and severe diseased fish (Fig 1F and 1H). The healthy spleen showed less melano-macrophage centers and the splenocytes were stained evenly with regular cell shape (Fig 1E).

Brain: Enlarged blood vessels were detected in the brain of diseased fish and were associated with infiltration of a large number of lymphocytes. The neurons were disordered and vacuolated, and had marginated nuclear chromatin in moderate and severe diseased fish (Fig 1J and 1K). Serious infected fish showed severe edema of cerebral cortex (Fig 1L). The healthy brain vessels were narrow and the healthy neurons were typical in arrangement and stain evenly (Fig 1I).

Kidney: Edema of the renal glomerulus was observed in affected kidney tissues. Lymphocyte infiltration and melano-macrophage centers were observed in and adjacent to the areas of diseased glomeruli. Hematopoietic tissues were also infiltrated with a large number of lymphocytes (S1B Fig). No melano-macrophage center was observed in healthy kidney, and the cells were stained evenly (S1A Fig).

Liver: Infiltration of lymphocytes and macrophages was showed in enlarged hepatic sinusoids. The infected hepatocytes showed a condensed and marginated nucleus (S1D Fig). In healthy liver, the hepatic sinusoids contained a large number of erythrocytes and the hepatocytes were typical in arrangement and stain evenly (S1C Fig).

Heart: The infected heart showed disorder and disruption of muscular trabeculae, and widespread vacuolation of myocardial cells showed marginated nuclear chromatin (S1F Fig). The healthy myocardial cells were stained evenly with regular cell shape (S1E Fig).

Gill: Primary lamellae of the infected gills were swollen and had widespread infiltration of inflammatory cells. The secondary lamellae showed congestion with a large number of blood cells. Meanwhile, swelling and necrosis of the diseased branchial epithelial cells was detected (S1H Fig). The healthy primary lamellae were narrow, and the secondary lamellae with one-layer epithelium were stained evenly (S1G Fig).

### Virus isolation and replication

**Fig 2:**
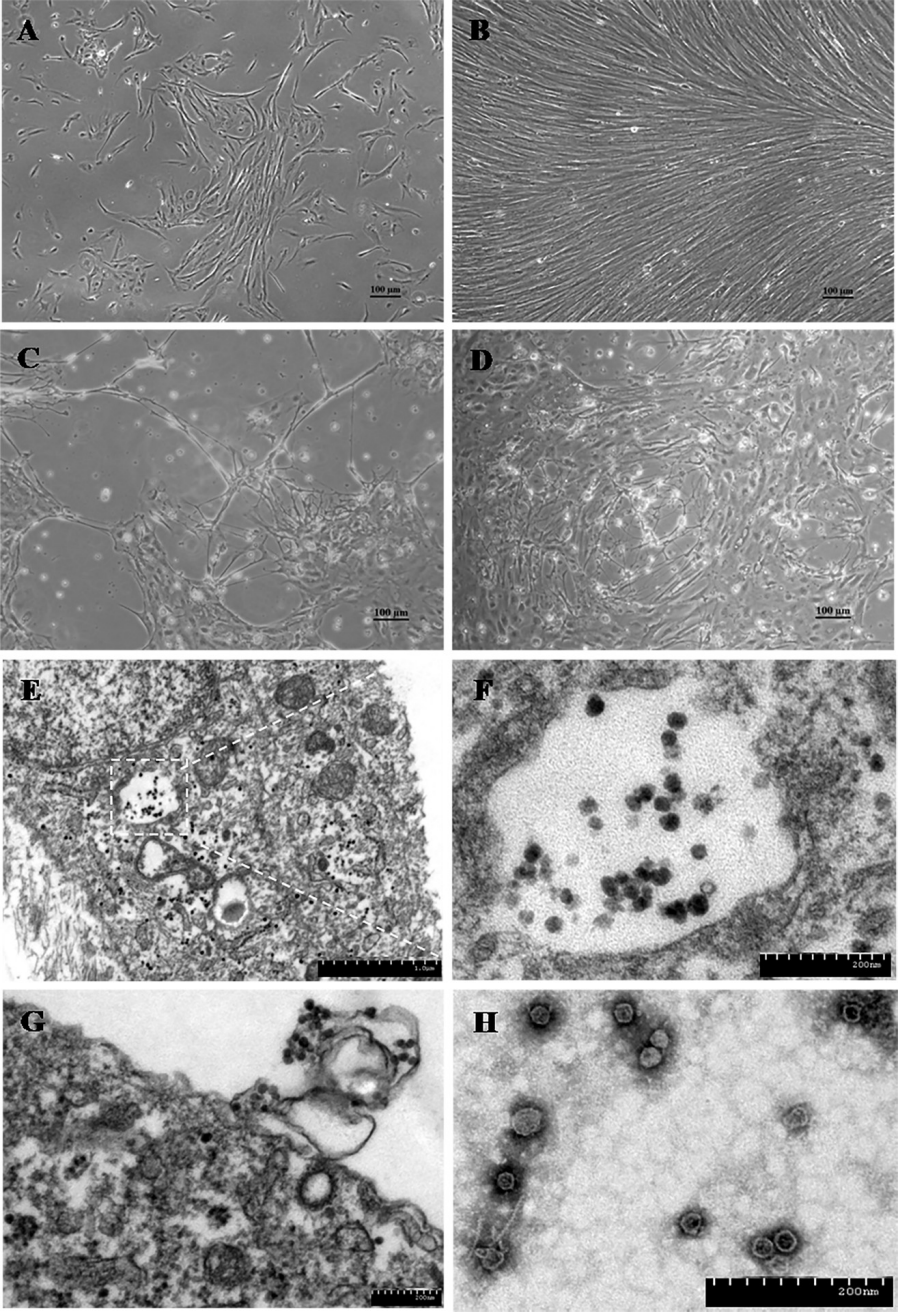
Morphology of the Tilapia brain cells (TiB) and cytopathic effect (CPE) induction on TiB induced by TiPV and transmission electron micrographs of the TiPV-infected TiB cells. (A) The TiB cells at passage 1, 10 days; (B) The TiB cells at passage 2, 3 days; (C) TiB cells infected with TiPV at passage 3 at 5 days post infection; (D) TiB cells infected with TiPV at passage 6 at 3 days post infection (Bar = 100 μm). (E) Virus particles existed in the cytoplasm and nuclei (white arrow), Nu: nucleus.(Bar, 1 μm); (F) High magnification of the region in the white rectangular box indicated in Panel A, virus particles aggregated in the cytoplasm (Bar, 200 nm); (G) The virus releasing at the plasma membrane of the TiB cell. (Bar, 200 nm); (H) Purified TiPV particles negatively stained with 2% phosphotungstic acid (Bar, 200nm).

On the initial isolation, tissue homogenates were inoculated onto TiB cells (Fig 2A and 2B). Cell morphology changed as early as 3 days post-infection (dpi), and cytopathic effect (CPE) was observed at 6 dpi (Fig 2C and 2D). Typical CPE included cell shrinkage, rounding, and cell fusion with cytoplasmic vacuolization. By 8 dpi, the cell monolayer was completely destroyed and TCID_50_ assays showed that virus titres reached 10^4.2±0.38^TCID_50_/ml at the 3^rd^ cell culture passage.

### Electron microscopy

**Fig 3:**
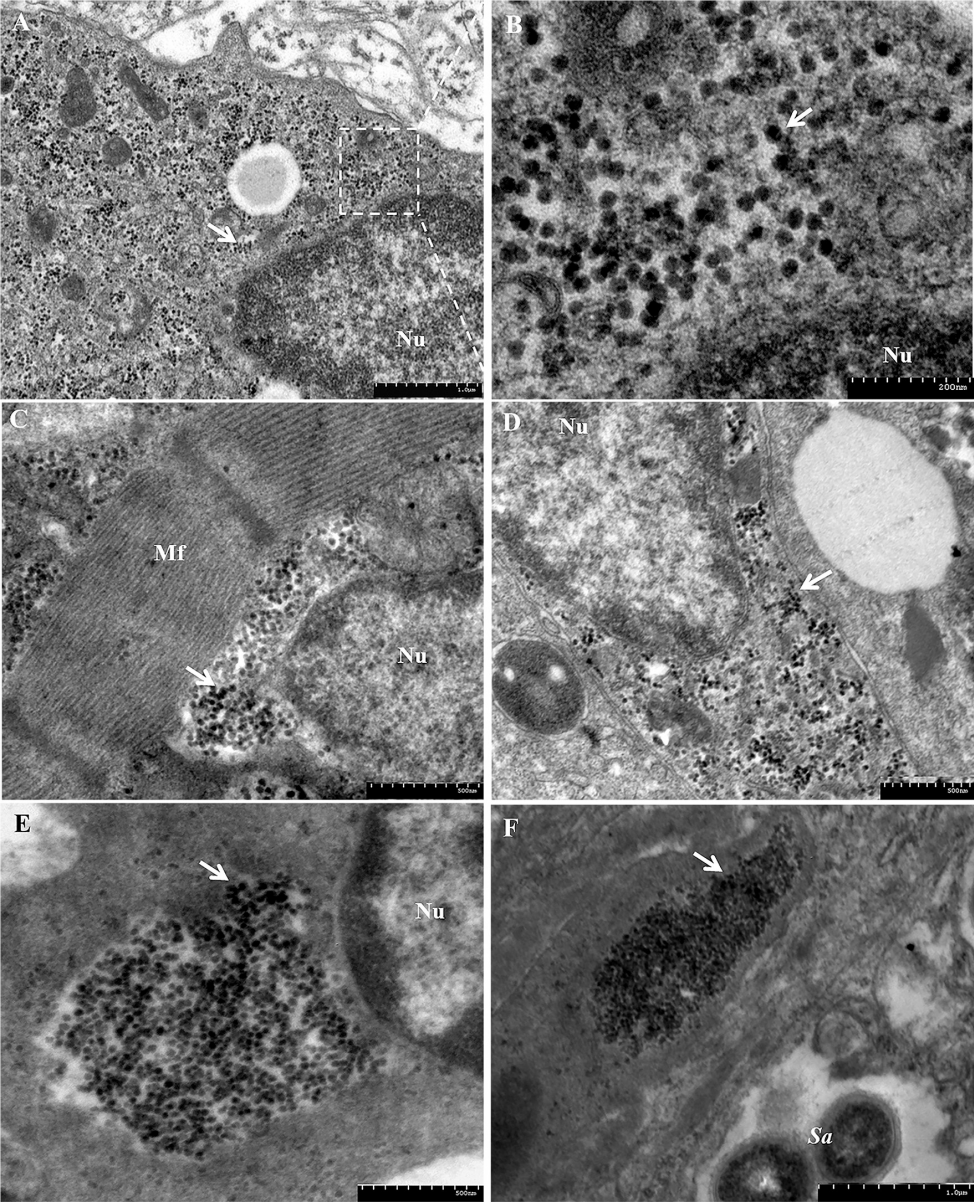
Transmission electron micrographs of the TiPV from the tissues of infected tilapia. (A) Brain. Large aggregates of parvovirus particles were present in the brain. Virus particles were located in the cytoplasm and nuclei (white arrow), Nu: nucleus (Bar, 1 μm); (B) High magnification of the region in the white rectangular box indicated in Panel A (Bar, 200 nm); (C) Heart. Large numbers of virus particles occupy the interfibrillar spaces, Mf: Myofibril (Bar, 500nm); (D) Eye. (Bar, 500nm); (E) Spleen. The mature virus clustered in the cytoplasm near the cell nucleus. (Bar, 500nm); (F) Spleen. Small amounts of bacterial pathogens (*Streptococcus agalactiae*) and virus co-infected the same spleen cell (bar, 1μm).

Ultrastructurally, the virus was primarily identified in cells of the heart, spleen, kidney, brain and gill, while eye and liver were shown to harbor the virus at apparently lower levels (Fig 3). Electron microscopy revealed large aggregates of parvovirus particles located in the cytoplasm and nuclei of cells. Virus induced lesions were also observed in sections of infected TiB cells, and showed vacuolation, karyolysis, chromatin condensation and margination. Some infected cells were completely lysed. Electron microscopic examination also revealed co-infection of the same spleen cell with virus and small amounts of the bacterial pathogen (*Streptococcus agalactiae*) (Fig 3F).

The virus particles in the TiB cells were dispersedly located in the nuclei or clustered in the cytoplasm near the cell nucleus or were observed releasing at the cell membrane (Fig 2E–2G). Both empty capsids and fully mature virions were observed following negative staining. The virions were spherical in shape with an inner nucleocapsid surrounded by capsids and non-enveloped, approximately 30 nm in diameter (Fig 2H).

### TiPV replication and distribution in naturally infected tilapia

**Fig 4:**
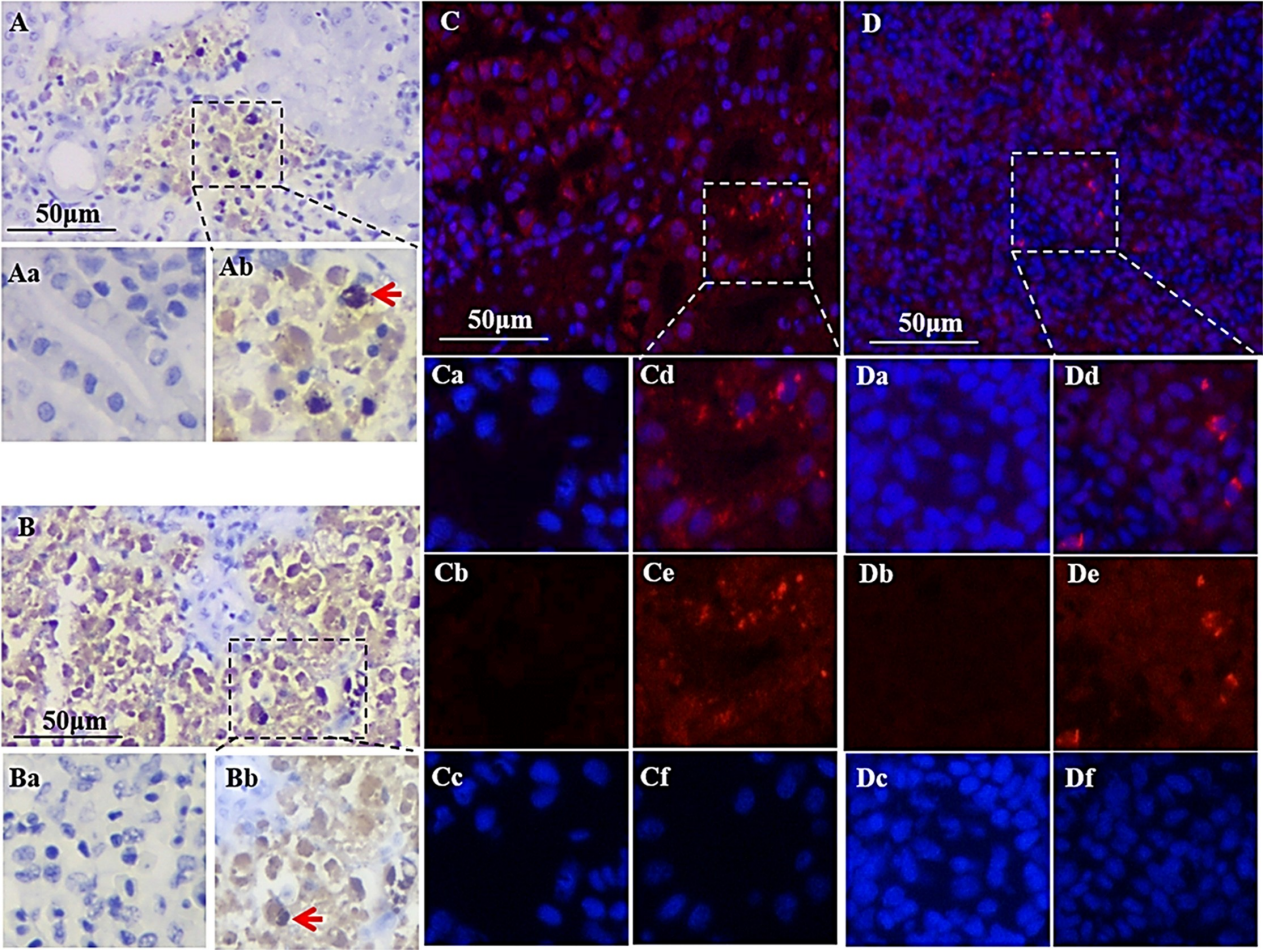
TiPV replication and viral protein expression in naturally infected tilapia. (A, B) *in situ* hybridization of the TiPV-infected kidney and spleen cells, respectively; (Ab, Bb) positive signals presented in kidney and spleen cells, respectively; (Aa, Ba) No signals presented in healthy kidney and spleen cells. Arrows indicate positive signals (bar = 20 mm); (C, D) Immunofluorescence assay of the TiPV infected kidney and spleen. The red color indicates the presence of viral protein. The nuclei are stained blue (bar = 50 mm); (Cd, Dd), (Ce, De) and (Cf, Df) Mock infected kidney and spleen cells, respectively; (Ca, Da), (Cb, Db), and (Cc, Dc) No fluorescence signals were observed in kidney and spleen cells of heathy fish.

The localization of viral mRNAs of TiPV in infected tilapia was investigated by *In situ* hybridization (ISH) and shown in Fig 4. Positive hybridization signals were observed in many tissue samples, including kidney, spleen, heart, brain, liver, gill and intestine. However, the intensity of hybridization signal varied among these organs. The strongest signals were observed in the kidney and spleen (Fig 4A and 4B). No signal was detected in negative control samples (Fig 4Aa and 4Ba).

### Identification of viral protein expression

The expression of viral protein was identified using indirect immunofluorescence (IFA) to determine the localization of TiPV in infected tilapia. As results show in Fig 4, strong signals could be detected in kidney and spleen tissues, and the TiPV infected cells were antigen-positive and exhibited bright red fluorescence in these tissues (Fig 4C and 4D). The specific fluorescence signals dispersed in the infected zone of cells cytoplasm were observed. In addition, no specific fluorescent signal was observed in uninfected tissues and the cell nuclei stained uniformly with DAPI (Fig 4Ca and 4Da).

### PCR assay and genome sequencing analysis

Amplification results showed that no PCR or RT-PCR positive samples were found for TLEV, VNN, iridovirus, or TiLV in naturally or artificially infected tilapia. However, PCR results showed several fragments ranging from 500 to 1000 base pair (bp) in length following amplification of the DNA template, but no fragments were obtained from the RNA template. Nucleotide sequence similarity searches using BLASTX found no significant matches with any other virus, while at the amino acid levels, sequence comparison did show a certain degree of similarity with *Parvoviridae* NS1 listed in the GenBank database. The genome sequence of NS1 revealed a novel parvovirus identified from diseased tilapia, which was subsequently named TiPV.

**Fig 5:**
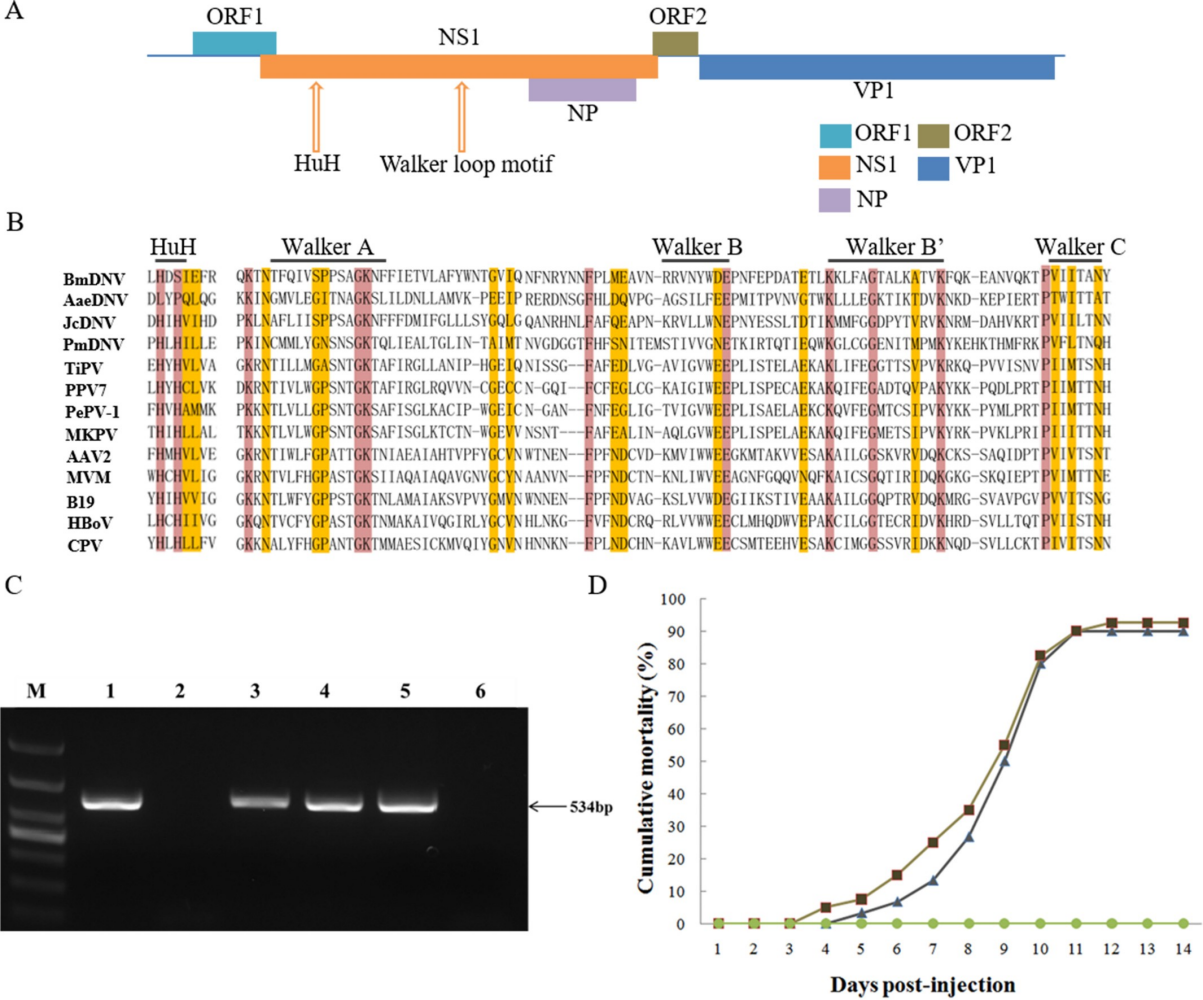
Characterization of the TiPV genome, PCR detection of TiPV in different samples and cumulative mortality of artificial infected tilapia. (A) The genome organization of TiPV. The NS1, NP, VP1 proteins, the alternative ORF1 and ORF2 are showed in different colors; (B) Alignment of conservative domains of NS1 proteins from different parvoviruses by Muscle package. The HuH (u indicates hydrophobic residues) and the walker loop motif, including A, B, B′ and C Walker box of helicase domains are marked; (C) Lane M: DL1000 bp DNA ladder; Lane 1: positive control; Lane 2: negative control; Lanes 3: the kidney tissues sample from natural infected tilapia; Lanes 4: TiB cell culture 3^rd^-passage viral supernatant; Lane 5: The artificial infected tilapia peritoneal injection with 0.5 ml of 0.22μm filtrate of diseased fish tissue homogenates; Lanes 6: the mock-infected tilapia; (D) Fish in the test group 1 (▲) were challenged by intraperitoneal injection with the 2^nd^-passage of viral supernatant (10^4.0^TCID_50_/ml) from cell culture; fish in test group 2 (■) were challenged by intraperitoneal injection with 0.5 ml filtrated supernatant homogenate from diseased fish tissues; and fish in the control group (●) were injected intraperitoneally with Dulbecco’s PBS.

Following amplification, a nearly complete genome (4269 bp) of TiPV was obtained, including 5' untranslated region (UTR) (208 bp), ORF1 (396bp), the complete NS1 sequence (1857 bp) and NS2 (504 bp) sequences, ORF2 (216 bp), the complete VP1 (1665 bp), and a partial 3' UTR (46 bp) (GenBank accession no. MT393593). The 5′end of assembled TiPV genome appears to be complete, however, genome assembly at the 3' end is lacking (Fig 5A).

The genome contained two major open reading frames (ORFs), with the left and right ORFs encoding NS1 and VP1, respectively. The TiPV NS1 (618 aa) contains a conserved replication initiator (endonuclease) motif as well as a helicase domain similar to other parvoviruses [13–15] (Fig 5B). The HuH motif (where u indicates a hydrophobic residue) was found located at aa 78–80. In addition, A Walker loop motif was also identified in the NS1 region at aa 264–360 in our sequences, including Walker A, B, B' and C boxes [16] (Fig 5B). However, An NP ORF was also identified in the same direction as NS1 located in a different reading frame. The predicted NP proteins for TiPV was 167 aa in length and BLASTP analysis of the protein determined that it had 50.0% aa homology with the function unknown NP protein of Cachavirus [17]. However, the NP coding sequence for TiPV was found to lack the translational start codon ATG.

The VP1 protein was 554 aa in length and significantly shorter than other members of the subfamily *Parvoviridae* (~700 aa). The complete of TiPV VP1 protein shares the highest amino acid sequence identity of 37.0% with *Parvoviridae* sp. (GenBank accession no. AUW34315) and the phospholipase A2 (PLA2) motif was not found in the N terminus of VP1 in TiPV. In addition, two ORFs (named ORF1 and ORF2) were identified within the left-hand ORF and between the NS1 and VP1, encoded a predicted 131-aa protein and 71 aa, respectively. Both of ORF1 and ORF2 were found that there were no significant matches with other parvoviruses by using BLASTP (Fig 5A).

**Fig 6:**
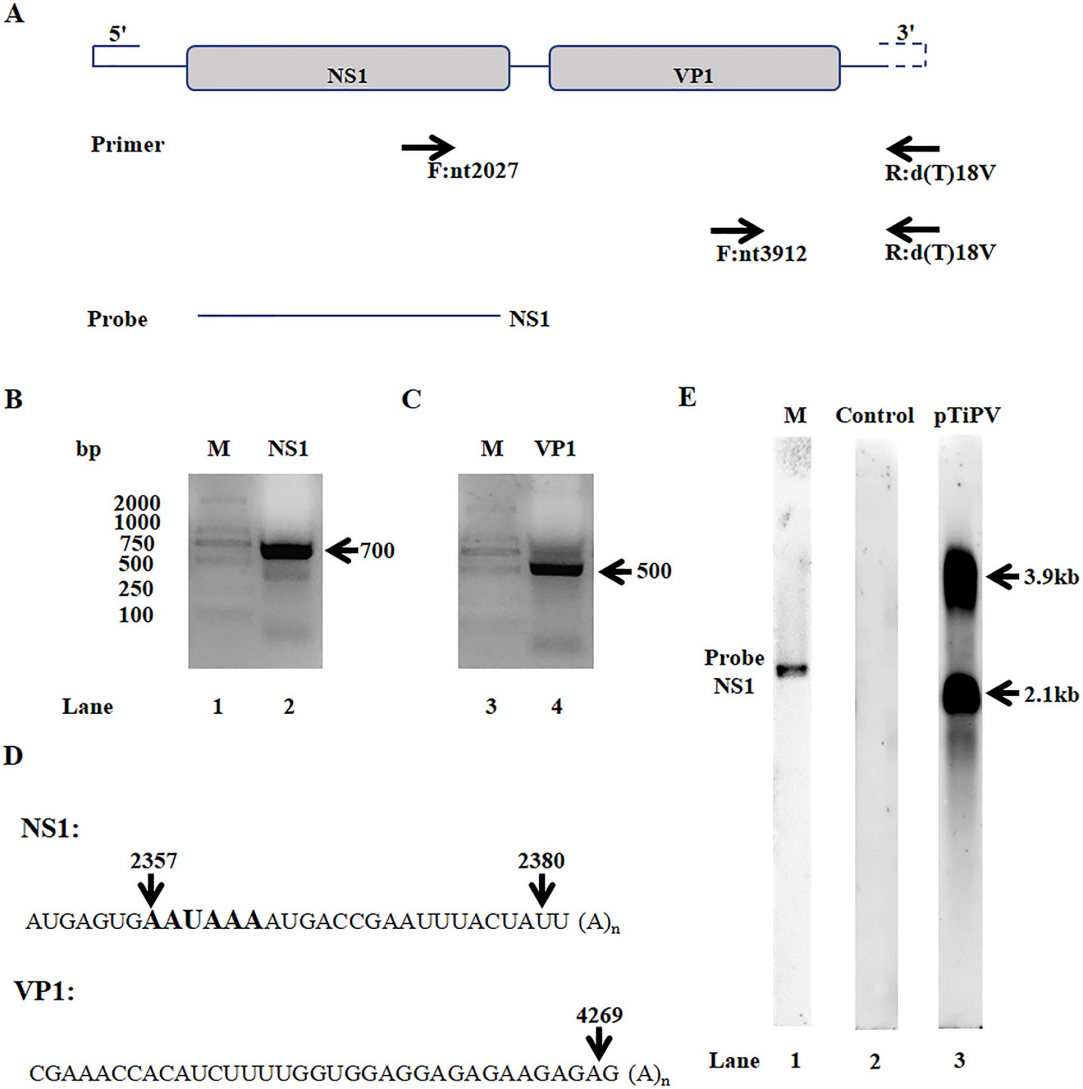
TiPV genome and transcription show its unique characterizations. (A) Assembled genome of TiPV. The major hypothesized ORFs, NS1 and VP1, including start and stop sites, and their orientation of them are both from 5′ end to 3′ end. The primers and probe below show their indicated locations in TiPV genome; (B, C) 3′ RACE analysis of NS1 and VP1. Total RNA was isolated from diseased kidney tissue and subjected to reverse transcription. The cDNA was then subjected to 3′ RACE analysis. Amplified DNA fragments were electrophoresed on 2% agarose gel and visualized using ethidium bromide staining; (D) The polyadenylation sites of TiPV. Sequencing of the PCR products from lanes 2 and 4 (B and C) show TiPV has two different polyadenylation sites; (E) Northern blot analysis. Total RNA was isolated from 293 cells transfected with pTiPV and empty vector, and used for Northern blot analysis. The blots were hybridized to probes NS1.

Since NP is a genus-specific gene, we focus mainly on the expression of NS1 and VP1. Both of them are highly conservative in phylogeny. To validate their mRNAs *in vivo*, RNA was extracted from the kidney tissue of diseased fish. Polyadenylated site of RNA utilizing was also detected by 3′ RACE. Specific PCR product was developed by using an anchor primer, NS1-specific primers (F2027) and VP1-specific primers (F3912), respectively (Fig 6B and 6C). Sequence analysis of these two bands revealed that they terminated at nt 2380 and nt 4269, respectively, suggesting that NS1 and VP1 mRNAs of TiPV are polyadenylated at nt 2380 and nt 4269. We also found that a consensus poly (A) signal (AATAAA) identified at nt 2357–2362 in the TiPV genome, which can be used for the (pA)p. However, no conventional poly (A) signal was identified upstream of nt 4269 (Fig 6D).

To verify NS1 and VP1 mRNA expression *in vitro*, we generated a nearly full-length infectious clone, which is named pTiPV and used it for the transcriptional profile. Northern blot analysis was performed using NS1 probe (Fig 6A). Hybridization of TiPV mRNA (extracted from pTiPV-transfected HEK-293T cells) with probe NS1 revealed two 3.9 kb and 2.1 kb bands (Fig 6E). The 3.9 kb band may correspond to the putative NS1 mRNA that is polyadenylated at nt 4269 (~3.9 kb), while the usage of polyadenylated site nt 2380 may generate a 2.1 kb band (Fig 6E).

Taken together, a nearly full-length sequence of TiPV was assembled and there were two major ORFs that encoded NS1 and VP1 proteins, respectively. We confirmed polyadenylated site of NS and VP by 3' RACE *in vivo*. Finally, the result of northern blot with probe NS1 also showed NS1 mRNAs have two different transcriptions polyadenylated at nt 2380 or nt 4269 *in vitro*.

PCR results for the non-structural protein 1 (NS1) fragment from samples of naturally infected tilapia and infected TiB cells were considered to be positive for parvovirus. The exact size of the cloned PCR fragment from TiPV was 534bp (Fig 5C).

### Quantification of TiPV distribution and viral loading in different tissues

**Fig 7:**
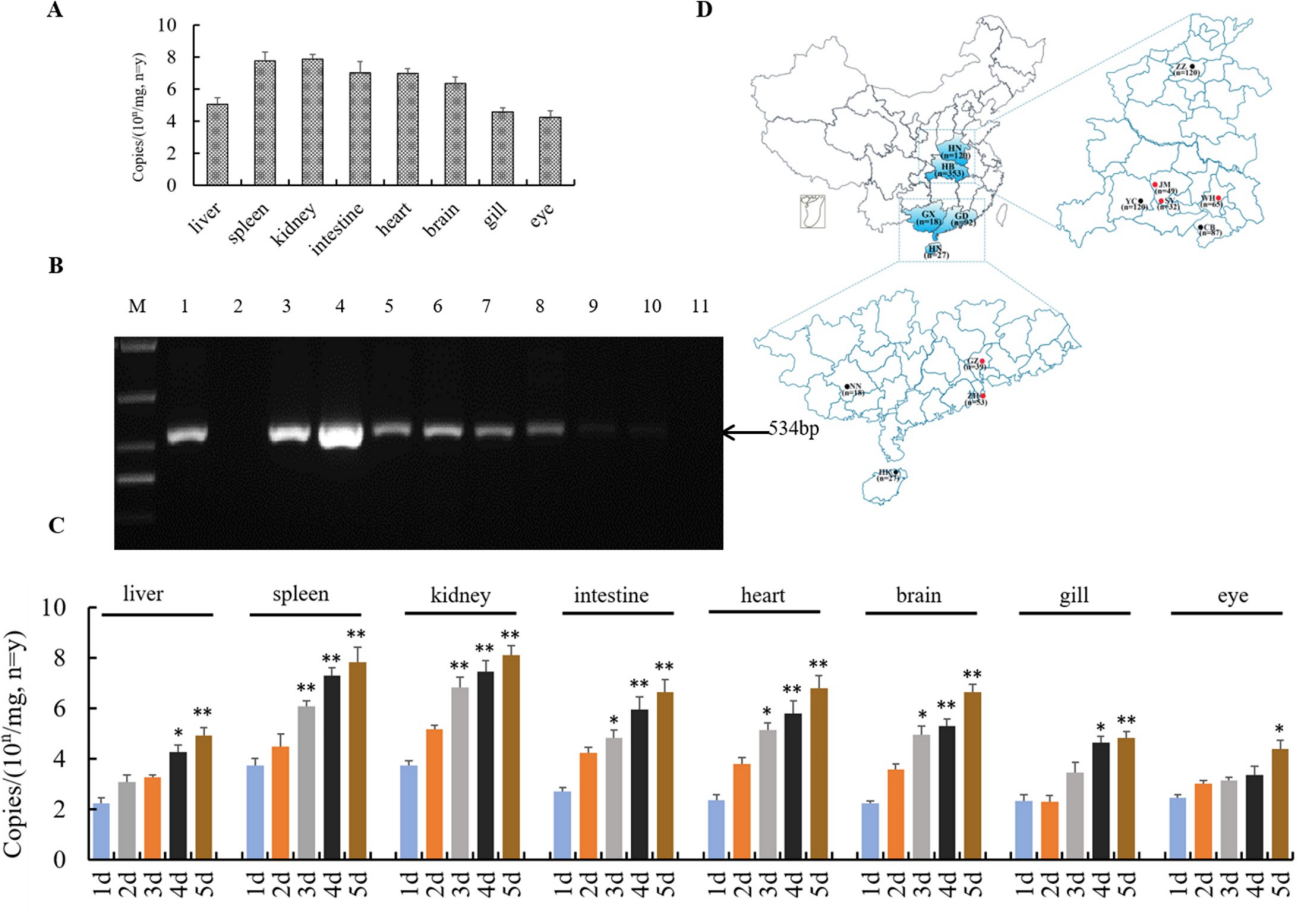
TiPV genome copies and distribution in affected tilapia and the prevalence of TiPV infection in different locations of China. (A) qPCR assay of TiPV genome copies in different tissues of the naturally infected tilapia. Each column represents the mean ± standard error of mean, with three independent replicates; (B) PCR detection of TiPV in naturally infected tilapia tissues samples. Lane M: DL1000 bp DNA ladder; Lane 1: positive control; Lane 2: negative control; Lanes 3: spleen; Lanes 4: kidney; Lanes 5: intestine; Lanes 6: heart; Lanes 7: brain; Lanes 8: liver; Lanes 9: gill; Lanes 10: eye; Lanes 11: muscle; (C) TiPV genome copies in different tissue samples of experimentally infected tilapia at different time point (1, 2, 3, 4, 5 dpi). Each column represents the mean ± standard error of mean, with three independent replicates; (D) Details of tilapia collection in Hubei province, Henan province, Guangdong province, Guangxi autonomous region and Hainan province. Circles: tilapia-sampling locations. Samples positive for parvovirus are identified in red, with negative in black.

The liver, spleen, kidney, heart, intestine, brain, eye, gill and muscle of naturally infected tilapia were collected and viral genome copies were detected using quantitative real-time PCR. The virus genome copies in infected kidney (3.5x10^7.32± 0.21^/ mg) and spleen (4.2x10^7.12± 0.35^/ mg) were higher than those from the intestine (4.3x10^6.37± 0.36^/ mg), heart (5.1x10^6.25± 0.27^/ mg), or brain (1.8x10^6.09± 0.24^/ mg). In samples of gill, liver and eye, genome copies were lower than above tissues and showed 1.8x10^4.32± 0.17^/ mg, 3.5x10^4.15± 0.26^/ mg and 2.8x10^3.78± 0.32^/ mg, respectively. No significant viral quantities were found in muscle samples or and known negative tissue samples (Fig 7A and 7B).

Quantitative real-time PCR of TiPV was also performed to estimate viral loads in the eight tissues (liver, spleen, kidney, heart, intestines, brain, eye, and gill) sampled at various time-points out to 5 days post-TiPV challenge. Results showed that the viral DNA could be quickly detected in the examined tissues of kidney and spleen at 1dpi with values of 3.2x10^3.22± 0.18^/ mg (kidney) and 3.5x10^3.18± 0.30^/ mg (spleen) and then continued to increase to 2.6x10^7.67± 0.34^/ mg and 3.4x10^7.28± 0.18^/ mg by 5 dpi in the TiPV challenged fish. The concentration of virus in heart, intestine, and brain tissues from 1 to 3 dpi were lower than spleen and kidney tissues, but again showed a steady increase reaching maximum values (3.6x10^6.24± 0.24^/ mg, 1.8x10^6.39± 0.15^/ mg, and 2.9x10^6.18± 0.21^/ mg for tissue, respectively) at 5dpi. The amount of TiPV was lower in gill, liver and eye tissue until 3 dpi, but maximum values (1.5x10^4.45± 0.18^/ mg, 4.2x10^4.28± 0.34^/ mg, and 3.6x10^3.82± 0.23^/ mg) were recorded at 5 dpi (Fig 7C).

### The prevalence of TiPV infection in different locations of China

Tissue samples from 610 tilapia were tested for TiPV by PCR targeting the NS1 gene regions. The fish were collected at water temperatures from 22°C to 32°C. Results showed that samples from six cities in three provinces were positive for TiPV. Of 82 samples collected in Hubei province, samples from Wuhan (42/65; 64.6%), Jingmen (30/49; 61.2%) and Shayang (10/32; 31.2%) were shown to be positive for TiPV. In Guangdong, the positive rates of TiPV were 23.1% (9/39) in Guangzhou and were 22.6% (12/53) in Zhuhai. Of 27 tilapia samples from Haikou of Hainan, 10 of these (37.0%) were positive for TiPV (Fig 7D). Multiple sequence alignment of these positive sequences showed identities of 92.5% (nt) ~ 99.1% (nt) with TiPV.

### Phylogenetic analysis and classification

**Fig 8:**
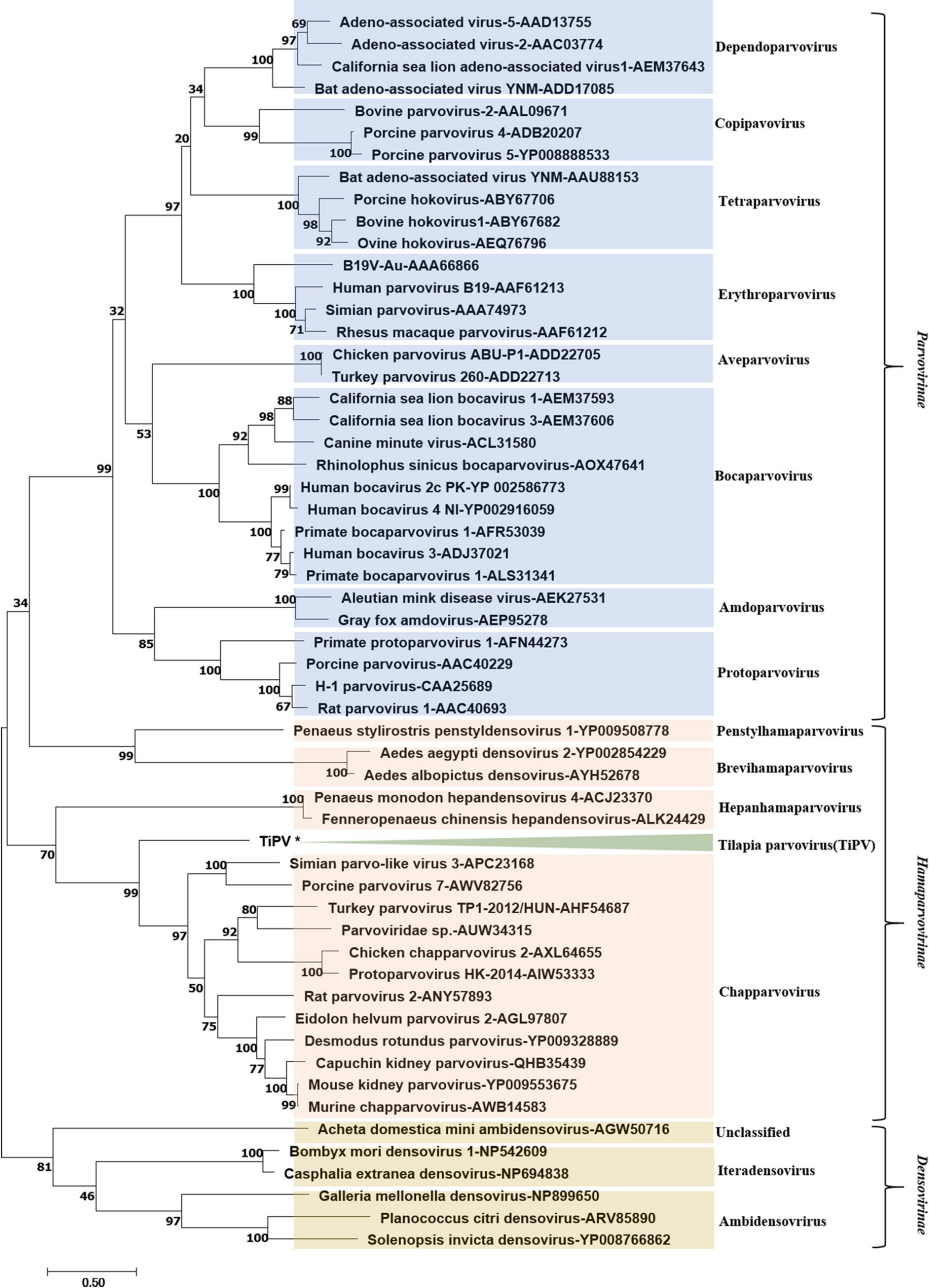
Phylogenetic analysis based on amino acid sequences of the non-structural 1 (NS1) protein from the family *Parvoviridae*. The phylogenetic tree is constructed based on the NS proteins of 55 representative parvoviruses using the Maximum Likelihood method with 500 bootstrap replicates. TiPV are indicated with shading and the number under the branch indicates the bootstrap values (values lower than 50 were hided). The scale bar means the genetic distance, number of substitutions per site.

A phylogenetic tree was constructed based on the completed non-structural (NS1) protein amino acid sequences of 55 representative viruses in the family *Parvoviridae* (Fig 8). The deduced TiPV NS1 protein has the highest similarity of 36.7% identical amino acid residues to the NS1 protein of Porcine parvovirus 7 (PPV7) [14]. Whereas similarity of 25–29% were found for the NS protein of Turkey parvovirus (TuPV) [18], Rat parvovirus 1 (RPV1) [15], Gray fox amdovirus (GFAV) [19] and human bocaviruses 1 (HBoV1) [20], and lower sequence similarity of 15–25% were found for Human parvovirus B19 (B19-Au) (AAA66866), Adeno-associated virus-2 (AAV2) (AAC03774), Bovine parvovirus-2 (BPV2) (AAL09671) and Bovine hokovirus 1 (ABY67682). In comparison with the Bombyx mori densovirus 1 (NP542609), identity was less than 9.4%. Phylogenetic analysis showed that TiPV is a novel parvovirus, and forms a separate branch in the proposed genus Chapparvovirus of *Parvoviridae* (Fig 8).

### Animal experiments

Healthy tilapia were challenged with purified virus derived from cell culture and exhibited ocular lesions and hemorrhagic symptoms that were similar to those found in naturally diseased fish at approximately 4–6 days post-infection (dpi). The mortality reached approximately 90% within 11 dpi (Fig 5D). The same symptoms and mortality rate were also observed for fish injected with tissue homogenates obtained from severely diseased fish. All fish from the mock-infected group remained asymptomatic. Samples from dead fish or fish from the challenged groups were PCR positive, while fish samples from the mock-infected group were negative (Fig 5C). Results clearly confirmed that TiPV isolated from infected tilapia and propagated in TiB cells, was indeed the etiologic agent of the observed disease in the tilapia farms.

## Discussion

Tilapia is one of the most important and fastest-growing aquaculture species worldwide. Due to the intensive farming techniques and routine movement of tilapia, emerging diseases have been documented [7–12]. One such emerging disease in farmed tilapia had recently spread in China, and this lethal disease is highly contagious and threatens the expansion of the industry. The present study describes the detection and characterization of a novel parvovirus, tentatively named Tilapia parvovirus (TiPV), as the causative agent of this emerging tilapia disease. This novel fish pathogen was identified following characterization of virus morphology, phylogenetic analysis, clinical symptoms, pathological changes, viral replication and protein expression, viral gene sequencing, and pathogen challenge experiments. These results showed that TiPV is a novel parvovirus pathogen, that is suggested to belong to a newly proposed genus, Chapparvovirus, that can cause massive mortality in tilapia. In recent years, numerous novel parvoviruses in Chapparvovirus have been reported primarily by high throughput sequencing [14–15, 17]. Among these groups, the novel parvoviruses have been largely obtained via fecal collection and swabs, and it remains unclear whether these viruses are disease-causing agents in these animals [13]. Recently however, two studies have claimed to demonstrate that a mouse kidney parvovirus (MKPV) [21] and a peafowl parvovirus 1 (PePV 1) [13] were identified from laboratory mice and dead peafowl during a disease outbreak. These findings imply that Chapparvovirus could be pathogenic and represents a potential disease threat to domestic species and wildlife [22]. However, the parvovirus morphology was absent in both infected tissue samples and *in vitro* in their reports. In our study, the virus was observed in cells of infected tilapia and TiB cells, which provides a basis for future study of the morphology of parvoviruses in the genus of Chapparvovirus.

Parvoviruses, which frequently infect animals through the oral-fecal-route, are small, non-enveloped icosahedral viruses with a capsid of 20-30nm in diameter and linear ssDNA genomes of 5kb containing two open reading frames that code for a non-structural protein (NS) and a capsid protein (VP) [23]. Based on their host range, the family *Parvoviridae* has been classified into two subfamilies: *Parvovirinae*, infecting vertebrates, and *Densovirinae*, infecting insects and other arthropods [24]. Parvoviruses have not been reported in teleost fish species. In this study, the TiPV NS1 was found to possess 9.4%-36.7% aa similarity to other parvoviruses and formed a distinct branch upon phylogenetic analysis, and supports our finding of a novel parvovirus TiPV infecting tilapia. Parvoviruses were thought to be species specific; however, studies of feline parvovirus (FPV) have determined that minimal sequence changes in the capsid region of the genome can have dramatic consequences for host range [25–26]. Parvoviruses are typically excreted in feces by homeothermic species and are extremely resistant to heat and desiccation. Such persistence in the environment can allow wide dissemination and further infection [27].

Many viral pathogens have been described as potential challenges to tilapia, including herpes-like tilapia larvae encephalitis virus (TLEV) [7], viral nervous necrosis (VNN) betanodavirus [8–9], iridovirus [10] and more recently the Tilapia Lake virus (TiLV) [11–12]. These viruses were not detected in this study in either naturally or artificially infected tilapia. Although *Streptococcus agalactiae* was confirmed in tissues from naturally infected fish as a co-infection with TiPV and may have contributed at some level to mortality, experimental challenges with the isolated virus demonstrated that TiPV is highly pathogenic without bacterial involvement. On the other hand, cohabitation infection may present a more natural way of transmission, a cohabitation challenge test should be needed for confirmation of the pathogenic agent in future investigations.

In addition to the outbreak and findings presented here, farmed tilapia suffering with similar clinical signs were sampled from Guangdong and Hainan provinces in China and were found to be positive for TiPV by PCR and sequencing (Fig 7D). This suggests an emerging outbreak of this disease may be spreading within the major tilapia culture regions within China, and could be linked to the environmental stability and highly contagious nature of parvoviruses [28].

Based on this work, TiPV appears to be a newly emerging pathogen for tilapia, and considering the extensive commercial production of tilapia worldwide [1, 2], it will be important to monitor stocks to prevent further spread of this pathogen. Results presented here provide a nearly complete molecular characterization of TiPV, including the two major open reading frames (ORFs), with the left and right ORFs encoding NS1 and VP1, respectively. These are the only regions exhibiting clear homology to those found in other parvovirus groups. Overlapping the NS, a predicted minor ORF (NP) was found and has no canonical start codon in our TiPV sequence, which is similar with porcine parvovirus 7 (PPV7) [14] and simian parvo-like virus 3 [SiPV3] (GenBank accession no. KT961660). This implies that in these viruses, splicing of the *rep* RNA is required for expression of the NP protein [22]. In addition, amplification of TiPV sequences from diseased fish and TiPV-infected cell cultures provides the basis for a fast and safe PCR-based diagnosis that could be implemented for screening, surveillance, and epidemiological studies.

Viruses of the family *Parvoviridae* usually have tightly controlled host ranges and tissue tropisms [28]. This is the first known report in teleost. Pathological electron microscopy analysis showed the virus to infect and destroy the heart as cardiomyopathy syndrome, and nervous tissues such as the brain and retina in susceptible fish. A tilapia brain cell line was established with the goal to serve for isolation of virus and to study the viral pathogenesis (Fig 2). The virus is able to propagate in this cell line, which allowed harvest and pathogen challenge by injection into healthy fish. Resulting disease manifested and clinical signs matched those from naturally infected tilapia. This will be more helpful to clearly confirm that TiPV, isolated from infected fish and propagated in TiB cells, was the etiologic agent of this disease.

In conclusion, the causative agent of a new emerging disease associated with massive mortality in farmed tilapia, in China was identified in this study as a novel parvovirus. This virus is tentatively named tilapia parvovirus (TiPV) based on morphology, phylogenetic analysis, clinical symptoms, pathology, and pathogen challenge experiments. TiPV appears to be an emerging viral pathogen in tilapia with implications for culture of this species in China and elsewhere. Further work is needed to confirm host restriction to tilapia, expand screening efforts, and develop methods to prevent or control TiPV disease.

## Materials and methods

### Fish

Moribund adult tilapia, 25–30 cm in length and 500–600 g in body weight, were collected from a tilapia cage-culture farm in Jinmen county of Hubei province in August 2015. The diseased fish were kept on ice or in oxygenated bags and then transferred to laboratory for diagnosis and pathogen determination. For animal experiment, healthy tilapia (18-25cm in length) were obtained from the breeding station of Yangtze River Fisheries Research Institute, and no history of this disease was recorded. The healthy fish were acclimated in recirculation rearing systems (2 m x 1.5 m x 1.5 m) with aerated water at a temperature of 28°C and were fed with commercial foods regularly for 1 week before experimental infection.

### Parasitology and bacteriology

The exterior mucus, gills and viscera of clinically diseased tilapia above were sampled for parasitic examination through light microscope. For bacteria isolation, liver and kidney tissue from moribund fish were inoculated onto brain heart infusion (BHI; Difco, USA) agar plates and incubated at 25°C for 10 days. Any colonies grown were identified using the Biolog Automatic Microbial Identification System (BAMIS, Biolog, USA).

### Histologic analysis

Tissue samples of liver, spleen, kidney, digestive tract, gill, heart and brain from diseased tilapia were fixed in 4% paraformaldehyde (PFA) for 24 hours at 4°C, and washed with Dulbecco’s phosphate-buffered saline (DPBS, Sigma, USA), dehydrated with 30% sucrose/DPBS, and embedded in optimum cutting temperature compound (OCT). Samples were cut (8 μm thick) at -20°C with cryostat (CM1950, Leica, Germany), stained with Hematoxylin-Eosin (HE) and examined by light microscopy with CCD picture system (DM2500, Leica, Germany) [29, 30].

### Cell cultures, Virus isolation and propagation

Tilapia brain cell line (TiB) was used to culture potential pathogens from diseased tilapia. TiB is a fibroblast cell line derived from the brain of Nile Tilapia (*Oreochromis niloticus*) and developed as previously described [31]. TiB cells were cultured in DMEM medium (Sigma-Aldrich, USA) with 10% FBS at 28°C. Heart, kidney, spleen and brain tissues collected from the diseased fish were homogenized aseptically with nine volumes of Dulbecco's PBS, freeze-thawed for 3 cycles (-80°C to room temperature) and centrifuged at 4,000 x g for 20min (Sigma-3K15, USA) at 4°C. The supernatant was filtered through a 0.22 μm filter (Nalgene, USA), and then 1 ml filtrate was inoculated onto a confluent monolayer of TiB cells in a 25 cm^2^ flask (Corning, USA). The same volume of cell culture medium DMEM was used for a mock infection in TiB cells as negative control. After adsorption of the material for 1 h at 28°C, 5 ml medium supplemented with 2% FBS was added to the flasks and cell cultures were incubated at 28°C. The cells were examined daily under an inverted phase contrast microscope (Nikon, Japan). When 90% cytopathic effect (CPE) was observed, the culture supernatant was harvested following a 3 times freeze-thaw cycle and centrifugation at 2880g for 20min to remove cell debris. Subculture of the isolated virus followed methods described above, and supernatant from TiB cells was stored at -80°C.Virus titer was determined using the standard 50% tissue culture infective dose (TCID_50_) method in a 96-well plate [32].

### Electron microscopy

Gill, heart, spleen, and kidney tissues collected from moribund fish were fixed in 2.5% glutaraldehyde and a 1% osmium tetroxide for post-fixation, then dehydrated, embed, cut and stained with 2% uranyl acetate and lead citrate as previously described [33–34]. Additionally, virus infected TiB cells were fixed and scraped from the flask and centrifuged at 1,000 x g for 10 min. The pellets were fixed and processed for electron microscopy as described above. For negative staining, a suspension from the infected cell culture was centrifuged at 4,000 x g for 30min (Sigma-3K15, USA) to remove cellular debris, then ultra-centrifuged at 141370 x g (Beckman Optima L-80XPRotor SW28,USA) for 2 h at 4°C. The remaining pellet was resuspended in 1 ml Dulbecco's PBS, and drops of the purified virus were negatively stained with 2% phosphotungstic acid (pH 7.0) for 2 min on Butvar-coated grids. All samples were then observed with a transmission electron microscope at 80 KV (Hitachi-7650, Japan) [35].

### *In situ* hybridization (ISH)

To investigate mRNA expression of TiPV in the infected tilapia, *in situ* hybridization was performed with a digoxigenin (DIG)-labelled probe for the TiPV NS1 gene sequence (GenBank Accession no. MT393593), that was synthesized as previously described [36]. The primer sequences are shown in Table 1 (TiPV-Fi/Ri). The tissue samples of infected tilapia were fixed in 4% paraformaldehyde (PFA, Sigma, USA) overnight at 4°C, dehydrated with 30% sucrose/ PBS and then embedded in optimum cutting temperature compound (OCT, Leica, Germany). Tissue samples were sectioned (8 um) at -20°C (Leica CM1950), and were rehydrated with PBS three times then labeled with the NS1 anti-DIG probe for overnight incubation at 65°C. The sections were then incubated with anti-DIG antibody (1:4000, Roche Diagonostics Gmbh, Germany) over night at 4°C, and then stained with NBT/BCIP (Roche Diagnostics, Germany). In addition, the nucleus was stained with hematoxylin. All the sections were examined by light microscopy (Leica DM2500, Germany).

**Table 1:** Primers used in present study.

Virus name	GeneBank accession number	Primer name	Primers (5’-3’)	PCR annealing temperature	Sequence number
Tilapia larvae encephalitis virus (TLEV)	───	TLEV-F	GAGACCAGAAAGTGCTTCTC	60ºC	170bp
		TLEV-R	TCGTGGGCCTTATCCCGCGT		
Viral Nervous Necrosis (VNN)	EU700416	VNN-F	ACCTCAATACACCCGCACGCTCCTC	55ºC	353bp
		VNN-R	CACGGTCAACATCTCCAGTCCCAAG		
Betanodavirus	───	F	CACAATGGTACGCAAAGGTG	55ºC	219bp
		R	CCGAGTTGAGAAGCGATCAG		
Iridovirus	KX185156.1	BIV-F	CGCAGTCAAGGCCTTGATGT	57ºC	585bp
		BIV-R	AAAGACCCGTTTTGCAGCAAAC		
Tilapia Lake Virus (TiLV)	KJ605629	TiLV-F	TATGCAGTACTTTCCCTGCC	55ºC	491bp
		TiLV-R	TTGCTCTGAGCAAGAGTACC		
Tilapia parvovirus Virus (TiPV)	───	TiPV-rF1	ACGGGGAAGACTGCGTTTATTAGAG	67ºC	───
		TiPV-rF2	CTTGGAGATGGTGTGAAAATGAACG	67ºC	
		TiPV-rR1	CGCCTTAGGTACTTTATACTTCTCGC	65ºC	
		TiPV-rR2	AACATCCCCCGTCTAAATCGGTCAC	69ºC	
		F2027	TATCGGCAGCGCCTGCACCACA	65ºC	───
		F3912	GCAAGACGCACATGGTTAGGCT	67ºC	
		TiPV-F	GAGATGGTGTGAAAATGAACGGG	59ºC	534bp
		TiPV-R	CTATCTCCTCGTTGCTCGGTGTATC		
		TiPV-Fq	GCACCACAGCTGAGTACAAC	56ºC	134bp
		TiPV-Rq	AACTGCTCGGCTATCTCCTC		
		TiPV-Fi	CACCTTTTCTGCCACCGATAG	57 ºC	505bp
		TiPV-Ri	CTCCCGCAAGTAAGCAAGATAGG		

### Indirect immunofluorescence assay (IFA)

Polyclonal mouse antibody raised against non-structural protein (NS1) of TiPV was prepared by ABclonal (Wuhan, China) and used as the primary antibody. IFA was performed according to the protocol described by Ma et al. [37] with slight modifications. Uninfected tissues samples and TiPV infected tissue samples were taken as negative controls and positive controls, respectively. These samples were fixed in 4% paraformaldehyde/PBS for 24 hours at 4°C, and washed with DPBS. The samples were cut at -20°C with cryostat and permeabilized with 0.25% TritonX-100, then blocked for 1 h at room temperature in DPBS. After washing three times with PBS, the samples were incubated with mouse anti-TiPV serum (1: 200) for 1 h at 37 º C, washed and incubated with Cy3-conugated secondary antibody (1:500, Beyotime Inc., PRC) for 1 h at room temperature. Samples were rinsed in PBS and incubated in 0.1 ug/mL 4',6-Diamidino-2-Phenylindole (DAPI) (1 ug/mL, Sigma, USA) for 5 min. Fluorescent images were captured using a Leica DM2500 fluorescent microscope with a Leica DFC420C camera at a magnification of 40x objective.

### Virus genome sequence amplification

To determine if this viral pathogen matched known viruses, all samples were screened using specific primers (Table 1) for herpes-like tilapia larvae encephalitis virus (TLEV) [7], viral nervous necrosis (VNN) betanodavirus [8–9], iridovirus [10] and Tilapia Lake virus (TiLV) [11–12].

Random-PCR and sequence analysis were used to identify the unknown virus using sequence independent single primer amplification (SISPA-PCR) [38]. Supernatants from fish samples were carefully collected, centrifuged to remove cellular debris, and processed through a 0.22um filter. The virus was enriched by ultracentrifugation (Beckman Coulter Optima L-80 XP Ultracentrifuge), and then degraded by 100 units of DNase I (Qiagen, Germany) and 10 ug/ml RNase A (Sigma-Aldrich, USA) to eliminate residual nucleic acid contaminants by incubation at 37°C for 1h. RNA and DNA were isolated immediately after nuclease treatment using Trizol (Sigma, USA) and DNA viral kits (Omega, Germany) according to the manufacturer’s instructions. PCR amplification for the unknown virus was evaluated according to the manuscript for Random-PCR methods [38]. Fragments of DNA and RNA template range from 500 to 1000 base pair (bp) in length were excised from the gel and purified using a Gel Extraction Kit (Promega, USA). The PCR and RT-PCR products ranged from 500 to 1000 base pair (bp) in length and were ligated into the PMD-19T vector using a TA cloning kit (Takara, Japan) then transformed into DH5a bacterial cells (Takara, Japan). Positive clones were picked and sequenced by Sangon Biotech (Shanghai, China). The 5′ and 3′ regions of the NS of parvovirus in the diseased tilapia was identified by performing rapid amplification of cDNA ends (RACE) PCR with the Clontech SMART cDNA synthesis kit (TaKaRa, Japan). To extend the 5′ region of the cDNA sequence, 5′ RACE was performed using a gene-specific primer (TiPV-rR1–rR2; Table 1) [30]. 3′ RACE was performed using an oligo (dT) adaptor primer with the TaKaRa RNA PCR Kit (TaKaRa, Japan) and was directly used as a template for two round PCR detection with the F2027 for NS1 or F3912 for VP1 and a reverse adaptor primer (TiPV-rF1–rF2; Table 1).

### Transfection and RNA extraction

HEK-293T cells were maintained in Dulbecco's modified Eagle's medium (DMEM, Invitrogen, USA) supplemented with 10% fetal bovine serum (FBS) in 5% CO_2_ at 37°C. Five micrograms of pTiPV was transfected into HEK-293T cells cultured on 60-mm dishes using Lipofectamine 2000 reagent (Invitrogen, USA), according to manufacturer's instructions. Total RNA was extracted at 48 h post transfection using TRIzol regent (Ambion, Foster City, CA).

### Northern blot analysis

Total RNA (30 μg) extracted from the pTiPV transfected HEK- 293T cells was subjected to electrophoresis in 1% agarose gel at constant voltage 28V for 12h. After that, the RNA was transferred onto a Hybond-N+ membrane (Amersham, Buckinghamshire, UK) and UV cross-linked. The blots were hybridized by using a DIG High Prime DNA Labeling and detection Starter kitⅡ(Roche, Germany), according to the manufacturer's instruction. Signals were detected by Gel Doc XR+ system (Bio-Rad, USA).

### PCR detection

Total DNA was extracted by DNAzol Reagent (Invitrogen, USA) according to the manufacturer’s recommended instructions from the purified virus obtained from infected TiB cells and tissue samples as described above. These were used as the templates for PCR and primer pairs were designed based on the non-structural protein (NS1) of parvoviruses in GenBank (GenBank accession no. MT393593) and the sequences for TiPV (Table 1). Primers were synthesized by Sangon Biotech (Shanghai, China) and PCR was performed with a primary denaturation step at 95°C for 5 min and then with 35 cycles starting with denaturation at 94°C for 30 s, annealing at 55°C for 30 s, extension at 72°C for 60s and a final extension at 72°C for 10 min. Amplified products were analyzed by electrophoresis using a 1.5% agarose gels and ethidium bromide staining. The expected PCR products at 534bp were excised, purified and sequenced as previously described [30].

### Quantitative real-time PCR

A real-time PCR assay was used for the detection and quantification of TiPV in infected tilapia tissue samples. Samples were collected from the 8±0.05 mg liver, spleen, kidney, heart, intestine, brain, eye, muscles and gill tissues of tilapia during 1–5 days post-infection with TiPV (intraperitoneal injection with 0.5 ml viral supernatant of the 2^nd^ passage with a titre of 10^4.0^ TCID_50_/ml). All the tissues were homogenized in lysis buffer, and virus genomic DNA was extracted using DNA viral kits (Omega, Germany) according to the manufacturer’s instructions. Real-time PCR reactions and quantitative analyses were performed primarily based on the previously described methods [37]. The primers used in this study are shown in Table 1(TiPV-Fq/Rq). Quantitative PCR was carried out in a 20 μl reaction volume using the following recipe: 2×SYBR Mix 10 μl, primers 0.25 μl each, DNA 1 μl, ddH_2_O 8.5 μl and the program was 94°C for 2 min, followed by 40 cycles of 94°C for 10 s, 60°C for 30 s. All samples from the infected group and the control group were tested three times by qPCR analysis. To determine the copy numbers of the TiPV DNA, a standard curve was generated with serial dilutions of synthesized DNA fragments of the TiPV NS1 gene. The virus genome copies per 1 mg tissues were calculated according to the Ct values following the manufacturer’s procedures (QIAGEN, Germany).

### Sample collections

To achieve a better understanding of the prevalence associated with TiPV, a total of 610 tilapia were collected from 10 different locations in Hubei (Jingmen, Shayang, Chibi, Yichang, Wuhan), Henan (Zhengzhou), Hainan (Haikou), Guangdong (Guangzhou, Zhuhai) and Guangxi (Nanning), China during 2015~2019. Tilapia were euthanized using MS-222 (Sigma-Aldrich, USA), and kidney and spleen were sampled and analyzed by PCR for TiPV detection. Fish were collected during different seasons, water temperatures and from ponds stocked with different sizes of tilapia. (Table 2, Fig 7C). Genomic DNA was extracted from tissues from each sample using a DNAzol Reagent (Invitrogen, USA) according to the manufacturer’s instructions and the positive samples were selected for sequencing by amplification the NS1 regions, which allowed investigation of genetic diversity of TiPV from different locations in this study.

**Table 2:** Origins of the TiPV isolates from different provinces used in this study.

Sampling location	Abbreviation	City	Date of collection	Temperature (°C)	Size (cm)	Positive samples No.
Jingmen	JM	Hubei	4 Aug. 2015	30	25~30	30
Shayang	SY		20 Aug. 2015	29	23~26	10
Chibi	CB		23 Jun. 2018	27	20~22	0
Yichang	YC		15 May 2019	22	14~17	0
Wuhan	WH		27 Jul. 2017	29	13~18	42
Zhengzhou	ZZ	Henan	25 Sep. 2019	28	18~23	0
Nanning	NN	Guangxi	2 Aug. 2017	32	20~26	0
Guangzhou	GZ	Guangdong	5 Sep. 2016	30	27~32	9
Zhuhai	ZH		12 May 2017	28	10~15	12
Haikou	HK	Hainan	15 Sep. 2015	32	15~20	10

### Phylogenetic analysis

Sequences were compared for similarity using BLAST (http://www.ncbi.nlm.nih.gov/blast). The deduced amino acid sequences were analyzed with the Expert Protein Analysis System (http://www.expasy.org). Multiple alignments of the deduced amino acid sequences were performed using Muscle package with default parameters. The phylogenetic tree containing the non-structural protein (NS1) sequences from other members of the family *Parvoviridae* was constructed with MEGA 6.0 using the Maximum-likelihood method with the substitution model of Poisson and rates of Gamma Distributed, while the support for the node was obtained with 500 bootstrap iterations.

### Animal experiments

Pathogen challenge experiments were performed in triplicate using healthy tilapia; 30 fish in test group 1 were challenged by intraperitoneal (IP) injection with 0.5 ml viral supernatant of the 2^nd^ passage with a titre of 10^4.0^ TCID_50_/ml, while 30 fish in the test group 2 were challenged by IP injection with 0.5 ml homogenate of diseased fish tissues prepared as prescribed above and filtered through a 0.22μm filter. Thirty fish in the control group were injected IP with 0.5 ml Dulbecco's PBS. All fish were held in tanks supplied with aerated water at 28°C during the infection experiment. Clinical signs and mortality were monitored daily, and samples from three moribund or dead fish per group (including controls) were randomly collected and processed for PCR to show presence or absence of the pathogen.

### Statistical analyses

Results were expressed as means ± standard deviation (SD) and all statistical analysis were done by using Student’s *t*-test (Prism v5, GraphPad Software) for determination of statistically significant differences which are indicated in graphs with asterisk symbols (*, *P<0*.*05*; **, *P<0*.*01*).

### Ethical statements

The study was performed in strict accordance with the Guide for the Care and Use of Laboratory Animals Monitoring Committee of Hubei Province, China, and the protocols (approval number: YFI2018-01) were approved by the Committee on the Ethics of Animal Experiments at the Yangtze River Fisheries Research Institute, Chinese Academy of Fishery Sciences. The tilapia was euthanized for 20–30 min in 1 mg/ml of MS-222 (Sigma, USA) before tissue collection.

## Supporting information

S1 Fig Pathological of kidney, liver, heart and gill. (A) Healthy kidney; (B) Diseased kidney, edematous renal glomerulus (red arrow), melano-macrophage centers (asterisk) and lymphocytes (white arrow) in affected kidney; (C) Healthy liver; (D) Diseased liver; lymphocytes (white arrow) and macrophages (black arrow) in diseased hepatic sinusoids; (E) Healthy heart; (F) Diseased heart, vacuolated mocardial cell (red arrow); (G) Healthy gill; (H) Diseased gill, inflammatory cells in primary lamellae (white arrow) and necrotic secondary branchial epithelial cells (red arrow). HE staining. Bar = 20um (A, B, D, F, G, H), 50um (C, E).
(TIF)

S1 Data Excel spreadsheet containing, in separate sheets, the underlying numerical data and statistical analysis for Figure panels 5D, 7A, 7C. (XLSX)
