# Supplementary figures and images for: Determination of a novel parvovirus pathogen associated with massive mortality in adult tilapia
Source: PLoS Pathog. 2020 Sep 24;16(9):e1008765. doi: 10.1371/journal.ppat.1008765 (PMC7588064; doi:10.1371/journal.ppat.1008765)

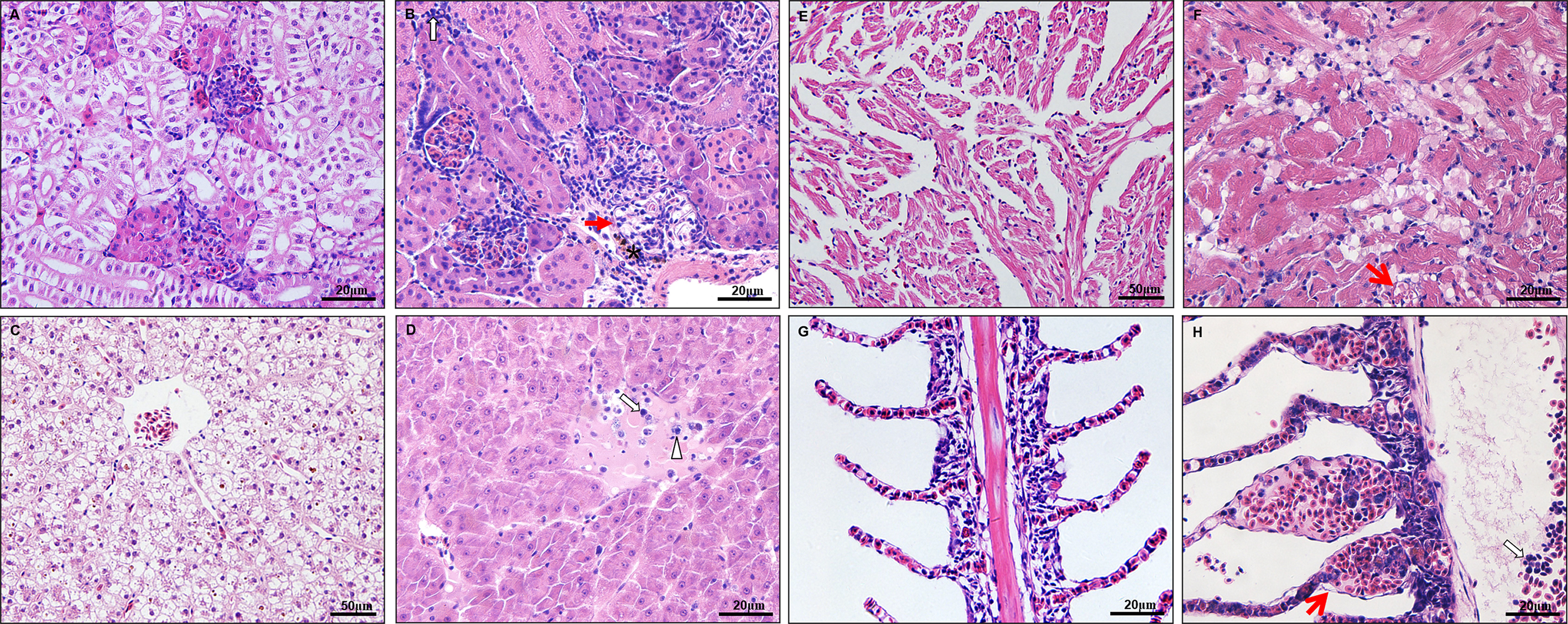

Supplement: S1 Fig — (A) Healthy kidney; (B) Diseased kidney, edematous renal glomerulus (red arrow), melano-macrophage centers (asterisk) and lymphocytes (white arrow) in affected kidney; (C) Healthy liver; (D) Diseased liver; lymphocytes (white arrow) and macrophages (black arrow) in diseased hepatic sinusoids; (E) Healthy heart; (F) Diseased heart, vacuolated mocardial cell (red arrow); (G) Healthy gill; (H) Diseased gill, inflammatory cells in primary lamellae (white arrow) and necrotic secondary branchial epithelial cells (red arrow). HE staining. Bar = 20um (A, B, D, F, G, H), 50um (C, E). (TIF) [file ppat.1008765.s001.tif]
